# Successful resection of a hypervascular paravertebral solitary fibrous tumor of pleura preserving the artery of Adamkiewicz, which was detected on preoperative angiography

**DOI:** 10.1186/s44215-023-00074-x

**Published:** 2023-09-06

**Authors:** Takehiko Manabe, Masataka Mori, Masatoshi Kanayama, Taiji Kuwata, Masaru Takenaka, Koji Kuroda, Satoshi Fukumitsu, Yu Murakami, Takatoshi Aoki, Toshiyuki Nakayama, Fumihiro Tanaka

**Affiliations:** 1 Second Department of Surgery, University of Occupational and Environmental Health, 1-1 Iseigaoka, Yahatanishi, Kitakyushu, 807-8555 Japan; 2 Department of Radiology, University of Occupational and Environmental Health, 1-1 Iseigaoka, Yahatanishi, Kitakyushu, 807-8555 Japan; 3 Department of Pathology, University of Occupational and Environmental Health, 1-1 Iseigaoka, Yahatanishi, Kitakyushu, 807-8555 Japan

**Keywords:** Solitary fibrous tumor of mediastinum, Hypervascular tumor, The artery of Adamkiewicz, Transcatheter arterial embolization

## Abstract

**Background:**

Solitary fibrous tumor of the pleura is rarely observed, accounting for 1% of all mediastinum tumors. There have been only a few reports of preoperative embolization for hypervascular tumors around the artery of Adamkiewicz (AKA). We report a rare case of solitary fibrous tumor for which transcatheter embolization was successfully performed before surgical resection.

**Case presentation:**

A 66-year-old woman with sudden-onset back pain was referred to our hospital for the evaluation of a left intrathoracic abnormal shadow on chest X-ray. Preoperative computed tomography (CT) showed a large, posterior mediastinal, paravertebral, and well-demarcated mass with high contrast enhancement and significant vascularization fed by the intercostal artery (ICA), measuring 8.1 × 7.6 × 6.4 cm. Therefore, solitary fibrous tumor, unicentric Castleman disease, or paraganglioma was included in the differential diagnosis. The patient underwent preoperative transcatheter arterial embolization followed by surgical extirpation. Thanks to the appropriate assessment of the anatomy, we could resect the tumor safely. The pathological diagnosis was solitary fibrous tumor of pleura.

**Conclusions:**

We recommend preoperative transcatheter arterial embolization (TAE) for hypervascular tumors close to the AKA that may require surgical removal; to reduce intraoperative hemorrhage, the AKA should be accurately detected during surgery.

## Background

Solitary fibrous tumor (SFT) is a fibroblastic mesenchymal tumor, and cases occurring in the mediastinum are relatively rare [1]. In general, large SFTs are often highly vascular, and preoperative embolization is sometimes required to control intraoperative bleeding [2]. However, due to the risk of complications (e.g., paraplegia and other spinal injury) from obstruction of the artery of Adamkiewicz (AKA), there are very few reports describing cases in which embolization was used in the treatment of intrathoracic paravertebral tumors. In the present case, a large progressive SFT of the pleura (SFTP) (maximum diameter, 81 mm) was so rich in blood flow that embolization of the feeding artery from the intercostal artery was considered necessary to ensure the safety of total removal. We herein report a case in which embolization followed by surgery was useful for preventing lethal hemorrhage and preserving the AKA during surgery.

## Case presentation

**Fig. 1:**
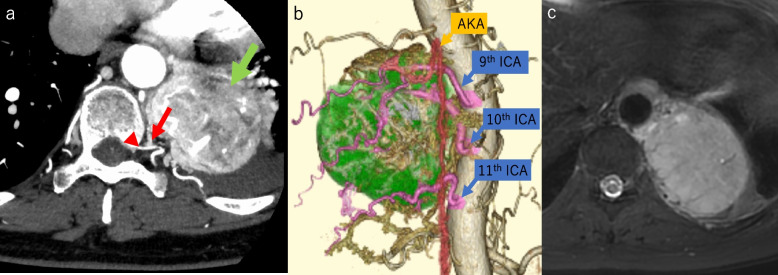
**a** Computed tomography revealed a large tumor (8.1 × 7.6 × 6.4) in the left posterior thorax that was in contact with the chest wall, mediastinum, and descending aorta. The T9th ICA (arrow) flowed into both the tumor (green arrow) and the AKA (arrowheads). **b** 3D‐CT image reconstruction showed the tumor occupying the left thorax (green) and the tumor‐feeding artery (the T9–T11th ICAs are colored in pink). The small arrow indicates the T9th ICA, and the arrowhead indicates the AKA. **c** T2-weighted MRI revealed a spherical tumor with high signal intensity

A 66-year-old woman was referred to our department because of back pain. Chest X-ray showed an abnormal mass in the left pericardial region, and computed tomography (CT) showed a large, posterior mediastinal, paravertebral, and well-demarcated mass with high contrast enhancement and significant vascularization fed by the intercostal artery (ICA), measuring 8.1 × 7.6 × 6.4 cm. A poor contrast area, suggesting necrosis or degeneration, was observed in the lesion (Fig. 1a). Chest magnetic resonance imaging (MRI) revealed that the lesion had a low signal intensity on T1-weighted images and slightly higher signal intensity on T2-weighted images (Fig. 1c). Radiologically, the differential diagnosis included solitary fibrous tumor, unicentric Castleman disease, or paraganglioma, and we considered that compression of the surrounding nerve had caused her back pain. A laboratory analysis showed a slightly high level of gamma-GTP (36 U/L). The patient’s tumor marker levels, including soluble interleukin-2 receptor (IL-2R), carcinoembryonic antigen (CEA), alpha-fetoprotein (AFP), and human chorionic gonadotropin (HCG), were within the normal ranges.

**Fig. 2:**
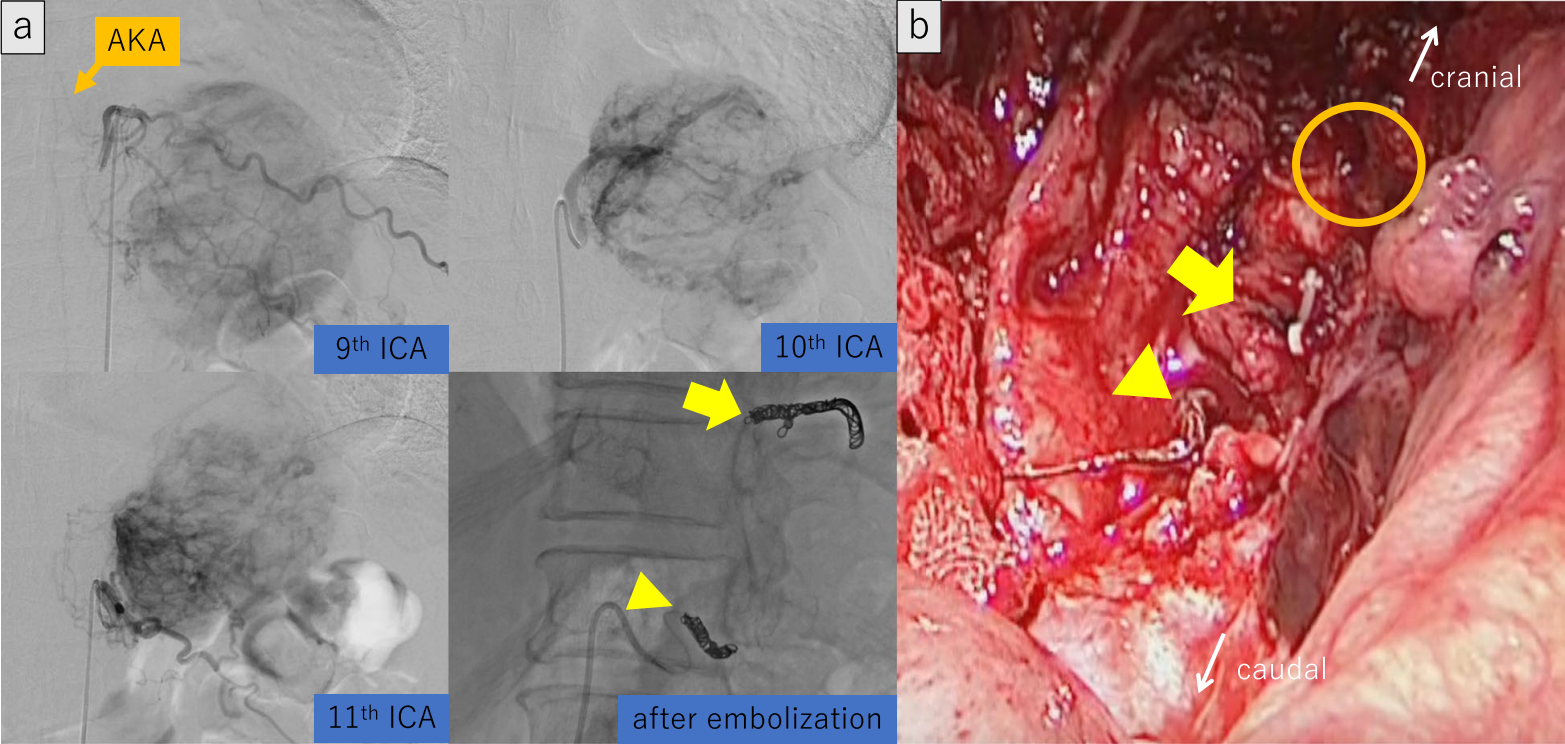
**a** Super‐selective catheterization of the T9, T10, and T11th ICA showed dense tumor staining. Embolization of the T10th (arrow) and T11th (arrowhead) ICAs was performed using coils and gelatin sponge, which resulted in reduced tumor staining. **b** Surgical image. We could detect the coils at the T10th (arrow) and T11th (arrowhead) ICAs and clipped these vessels at the proximal areas. In addition, we treated the vessels in peripheral area of T9th ICA and preserved its root that branches off the AKA (round)

**Fig. 3:**
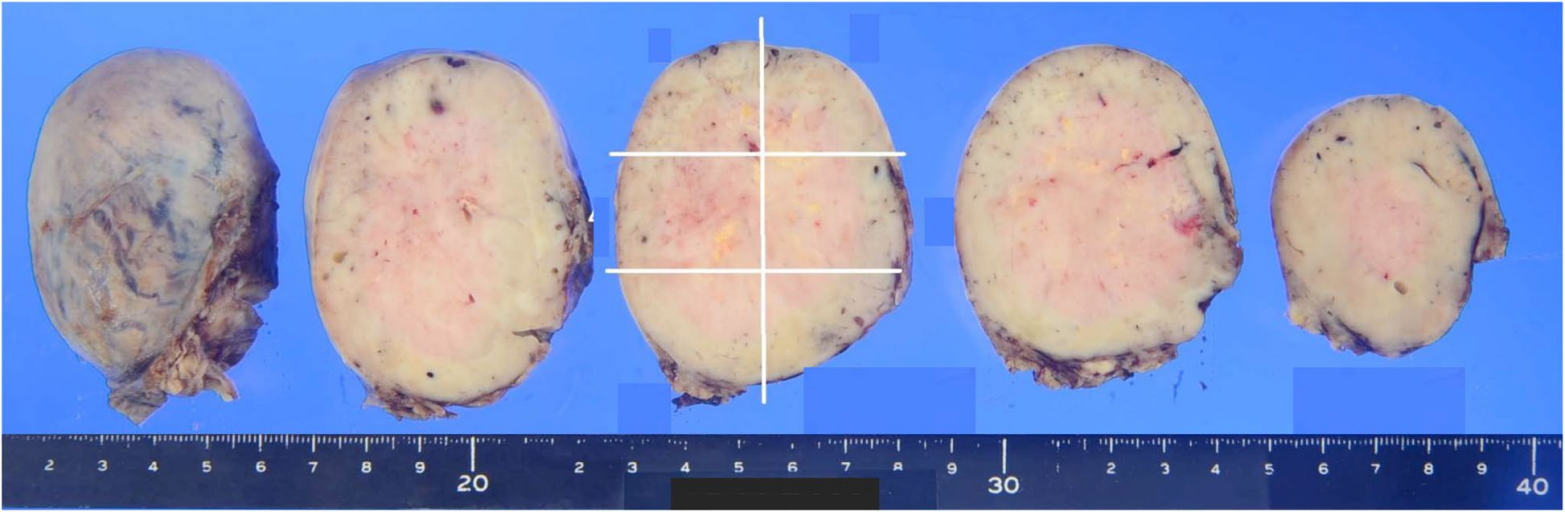
Macroscopically, the lesion was 8.1 cm in diameter. The cut surface of the lesion revealed a whitish-brown, elastic, hard mass

**Fig. 4:**
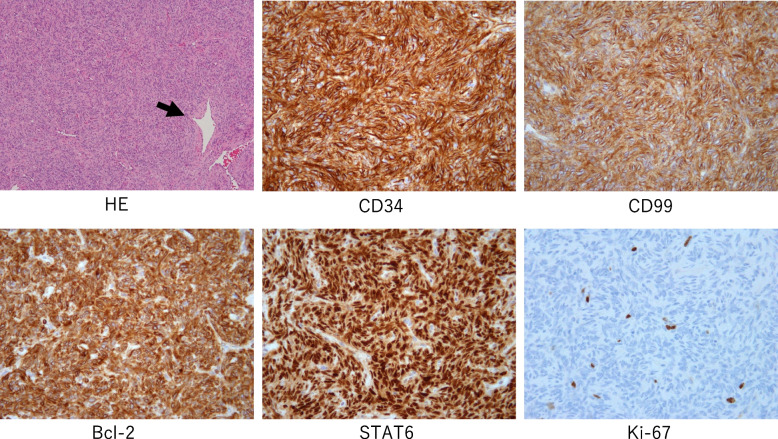
Microscopically, hematoxylin and eosin staining of the tumor showed disordered arrangement of spindle cells and the classical “staghorn” appearance with intense vascularity (arrow). Immunohistochemistry revealed that these cells were diffusely positive for CD34, CD99, Bcl-2, and STAT6 and negative for synaptophysin and chromogranin A, confirming a solitary fibrous tumor. The tumor had one mitosis per ten high-power fields, and the Ki-67 proliferation index was 3.0%, which was consistent with a low-grade tumor. All figures are shown at 40 × magnification

Because of the large size and the abundant blood supply to the tumor, we planned transcatheter arterial embolization (TAE) preoperatively, followed by surgical resection of the tumor. Angiography revealed that the tumor was supplied by branches arising from the left ICA between T9 and T12th, whereas the AKA also branched from the T9th ICA. The main feeders of the tumor were vessels from T9, 10, and 11th ICA. Furthermore, during the angiography of T12th ICA, the T11th ICA artery was visualized through the collateral return tract, and a portion of the tumor was also retrogradely stained. Super-selective catheterization and embolization of the T10 and T11th ICAs were performed using coils and gelatin sponge, and T12th ICA was embolized by gelatin sponge (Fig. 2a). Two days after the angiography, left posterior thoracotomy through the eighth intercostal space was performed to resect the tumor. During surgery, we observed a large well-demarcated mass around the T9–T11th vertebral body with elastic hardness. The tumor did not invade the surrounding structures (e.g., the diaphragm, pericardium, and lung) and could be safely resected without life-threatening bleeding. The total blood loss was 1330 ml, and she required blood transfusion of 840 ml of red blood cells and 720 ml of fresh-frozen plasma. Furthermore, we could successfully preserve AKA and cut the feeders branched from between the T9 and T11th ICAs by grossly detecting the coils placed before surgery (Fig. 2b). As resecting the vessels in order from the caudal part, the lesion was gradually separated from the important structures, such as the AKA. In addition, we used the motor-evoked potential (MEP) to evaluate the spinal cord injury and confirmed that blood flow was maintained in both lower extremities after resection of the tumor. The tumor was 8.1 × 7.6 × 6.4 cm with whitish-brown discoloration (Fig. 3). Histologically, the tumor had a fibrous capsule and consisted of monotonous spindle cell proliferation without atypia. Immunohistochemical staining demonstrated that the tumor cells were positive for CD34, CD99, Bcl-2, and STAT6. The tumor had one mitosis per ten high-power fields, and the Ki-67 proliferation index was 3.0%, consistent with being benign (Fig. 4). No other malignancy was identified.

Based on these findings, the pathological diagnosis of SFTP was made. The patient was discharged without any postoperative complications and was followed up for 6 months after surgery without any recurrence.

### Discussion and conclusions

Solitary fibrous tumor of pleura (SFTP) is a mesenchymal neoplasm of fibrous origin. About 80% of these tumors originate in the visceral pleura, and 20% arise from the parietal pleura. Tumors of more than 8 cm in size are more likely to have a parietal pleural origin and have a vascular pedicle [3]. The age of onset of SFTs is around 50 to 60 years, and the male-to-female ratio appears to be almost equal. The fifth WHO classification published in April 2020 subdivided SFT into three categories: benign (locally aggressive), malignant, and NOS (rarely metastasizing) [2]. SFT arises at various sites, and meningeal SFT, previously known as hemangiopericytoma (HPC), is a rare form of extra-pleural SFT that is derived from the smooth muscle pericytes surrounding the intraparenchymal microvasculature [4]. SFTs and HPCs are currently considered to be the same entity at two opposite ends of the same histologic spectrum rather than the strict “benign or malignant” dichotomy that was used for decades [2]. The present case was considered to be the HPC based on the conventional classification according to its immunohistological features, including a “staghorn” appearance.

Concerning its treatment, surgical removal is the first choice for local disease, with 10-year survival rates reported to be between 54 and 89% after complete surgical resection with clear margins [5]. Our decision to perform the resection after embolization was to reduce the risk of uncontrollable bleeding and prevent injury to the AKA. In the present case, because the preoperative CT and MRI revealed the tumor had extremely hypervascularity and localized around the T9–12th vertebral body, we conducted angiography in order to describe the feeding artery and clarify the relationship between the tumor and AKA, revealing that one of the main feeders of the tumor flowed into the spine area. In some studies involving intrathoracic hypervascular tumors that interrupt the vascularity, the use of embolization may decrease the amount of intraoperative bleeding and improve the safety of the procedure [1, 6, 7]. Kelin Yao et al. reported the case in which embolization of the hepatic artery and the phrenic artery was useful for preventing hemorrhage during surgery for SFTP [1]. However, our search of the relevant literature revealed no cases of SFTP in which transcatheter embolization was used for vessels around the AKA. To prevent critical hemorrhage during surgery and postoperative paralysis due to damage of the AKA, we performed the super-selective embolization of the ICAs between T10 and T12, avoiding for occlusion of the T9 vessels that flow into both the AKA and the tumor. Furthermore, during surgery, we could easily detect the coils and find the blood flow of the AKA; thus, these vessels could be safely cut at the proximal area. The detailed preoperative analysis of the vessels and occlusion by visible coils was useful for planning the surgical strategy. In addition, indocyanine green (ICG) imaging has been widespread use to check the course of each intercostal artery. There are some reports that mention the usefulness of ICG for detection of travelling of intercostal artery and evaluation of arteriovenous fistula (AVF) during surgery especially in the field neurosurgery. On the other hand, Kienzler, Schoepf, Marbacher, Diepers, Remonda, and Fandino reported that, in one case of AVF, intraoperative DSA showed failure of fistula occlusion, which was not visible with ICG angiography, leading to repositioning of the clip [8]. In this way, although ICG imaging for the evaluation of intercostal artery is not yet a well-established method, its development for accurate identification as a simple method and accumulation of those cases is desirable.

We herein report an extremely rare case of an SFTP located close to the AKA, which was successfully treated with preoperative embolization and surgical resection. Coil embolization of the feeding artery should be considered as a possible strategy.

## Data Availability

Not applicable.

## Abbreviations

AKA: Artery of Adamkiewicz
CT: Computed tomography
ICA: Intercostal artery
CEA: Including carcinoembryonic antigen
AFP: Alpha-fetoprotein
TAE: Transcatheter arterial embolization
HCG: Human chorionic gonadotropin
IL-2R: Interleukin-2 receptor
MEP: Motor-evoked potential
MRI: Magnetic resonance imaging
ICG: Indocyanine green
